# Risk factors of radial artery occlusion following via transradial coronary catheterization

**DOI:** 10.1186/s13019-026-04608-6

**Published:** 2026-07-29

**Authors:** Bing Xie, Yi Teng, Hui Feng

**Affiliations:** Department of Cardiology, The Affiliated Hospital of Xuzhou Medical University, 99 Huaihai West Road, Xuzhou, 221004 Jiangsu China

**Keywords:** Radial artery occlusion, Transradial coronary angiography and intervention, Risk factors, Prediction nomogram

## Abstract

**Objectives:**

This study aimed to identify the independent risk factors associated with radial artery occlusion (RAO) after right transradial coronary angiography (CAG) or percutaneous coronary intervention (PCI), and construct a visualized nomogram for individualized RAO risk prediction.

**Methods:**

This single-center retrospective study enrolled 561 patients with suspected angina pectoris undergoing right transradial CAG/PCI from December 2023 to June 2024; all patients received a uniform 6F radial sheath. Radial artery patency was evaluated 24 h post-procedure via modified reverse Barbeau test (MRBT). Patients were divided into RAO group (n=49) and non-RAO group (n=512). Univariate logistic regression screened candidate variables (screen threshold p<0.1), and backward stepwise multivariate logistic regression was used to identify independent predictors. A nomogram was established based on regression coefficients of independent predictors. Model performance was assessed by ROC curve (AUC), calibration curve combined with Hosmer-Lemeshow (HL) test, and decision curve analysis (DCA).

**Results:**

The overall incidence of RAO was 8.7%. Multivariable logistic regression identified four independent risk factors for RAO: prior coronary artery disease, multiple puncture attempts, puncture duration >15 min and radial arterial spasm. The AUC of the prediction model was 0.754 (95%CI 0.671–0.837). The calibration curve showed favorable consistency between predicted and actual RAO incidence, with HL test p=0.536. DCA verified reliable clinical net benefit of the nomogram for risk stratification.

**Conclusions:**

Four independent predictors of post-transradial RAO were confirmed:prior coronary artery disease, multiple puncture attempts, puncture duration>15min and radial arterial spasm. The nomogram built on these four factors exhibited satisfactory discrimination, calibration and clinical utility. This tool can help clinicians screen high-risk patients and deliver targeted preventive interventions, while avoiding unnecessary prophylaxis for low-risk individuals. Further prospective external validation of this model is required.

**Supplementary Information:**

The online version contains supplementary material available at https://doi.org/10.1186/s13019-026-04608-6.

## Introduction

Radial artery occlusion (RAO) represents the most common complication following transradial catheterization (TRC), a widely preferred approach for coronary angiography (CAG) or percutaneous coronary intervention (PCI) due to its advantages over transfemoral access, including improved patient comfort, earlier mobilization, and reduced bleeding risk [1]. The incidence of RAO ranges from 5% to 8% across clinical centers and may exceed 10% in certain settings [2, 3], with asymptomatic cases predominating. However, symptomatic RAO may manifest as pain, paresthesia, or impaired arm function. In rare cases with insufficient collateral circulation, ischemic complications may occur, primarily due to anatomical variations [4].

The pathogenesis of RAO is thought to involve endothelial damage during catheterization, followed by thrombus formation secondary to decreased blood flow, highlighting the interplay between mechanical injury and prothrombotic states [5, 6]. Despite its often-asymptomatic presentation, RAO remains clinically important because it may limit future use of the radial artery for repeat transradial procedures, coronary artery bypass grafting, or hemodialysis access [4, 7]. However, predictors of RAO remain incompletely understood and evidence is inconsistent, leading to a persistent lack of simple, clinically applicable tools for early risk prediction.

Therefore, this study aimed to identify independent RAO risk factors after right transradial CAG/PCI,establish an easy-to-use visualized nomogram prediction model for individualized preoperative RAO risk stratification, and verify its discrimination, calibration and clinical net benefit to provide a quantitative reference for clinicians to implement targeted preventive interventions for high-risk patients.

## Materials and methods

### Study subjects

**Fig. 1:**
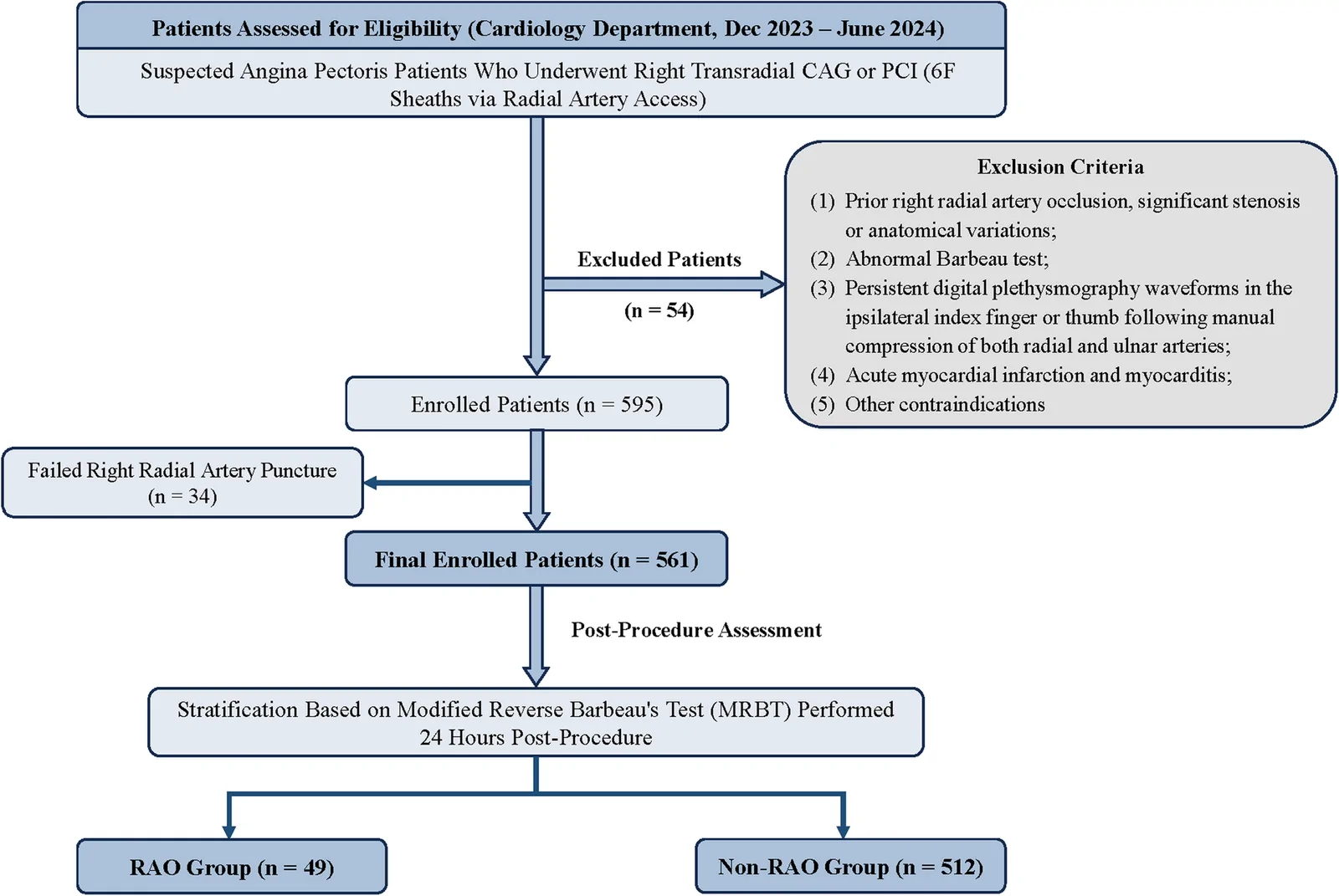
Study flowchart

Herein, a retrospective study was conducted to evaluate predictors of RAO in patients with suspected angina pectoris who underwent right transradial CAG or PCI in the cardiology department from December 2023 and June 2024.All procedures utilized 6F sheaths via the right radial artery access. The exclusion criteria included: (1) prior right radial artery occlusion, significant stenosis, or anatomical variation; (2) abnormal Barbeau test results; (3) persistent digital plethysmography waveforms in the ipsilateral index finger or thumb following manual compression of both radial and ulnar arteries; (4) failed right radial artery puncture; (5) acute myocardial infarction or myocarditis; or (6) other contraindications. Upon the application of the exclusion criteria, a total of 561 patients were ultimately enrolled in the study. Based on the modified reverse Barbeau’s test (MRBT) performed 24 hours post-procedure, the patients enrolled were stratified into RAO(n=49) and non-RAO (n=512) groups (Fig. 1). The study involving human participants was conducted in accordance with the Helsinki Declaration and was approved by the Ethics Committee of the Affiliated Hospital of Xuzhou Medical University (Approval No: XYFY2025-KL408-01).

### Modified reverse barbeau’s test

**Fig. 2:**
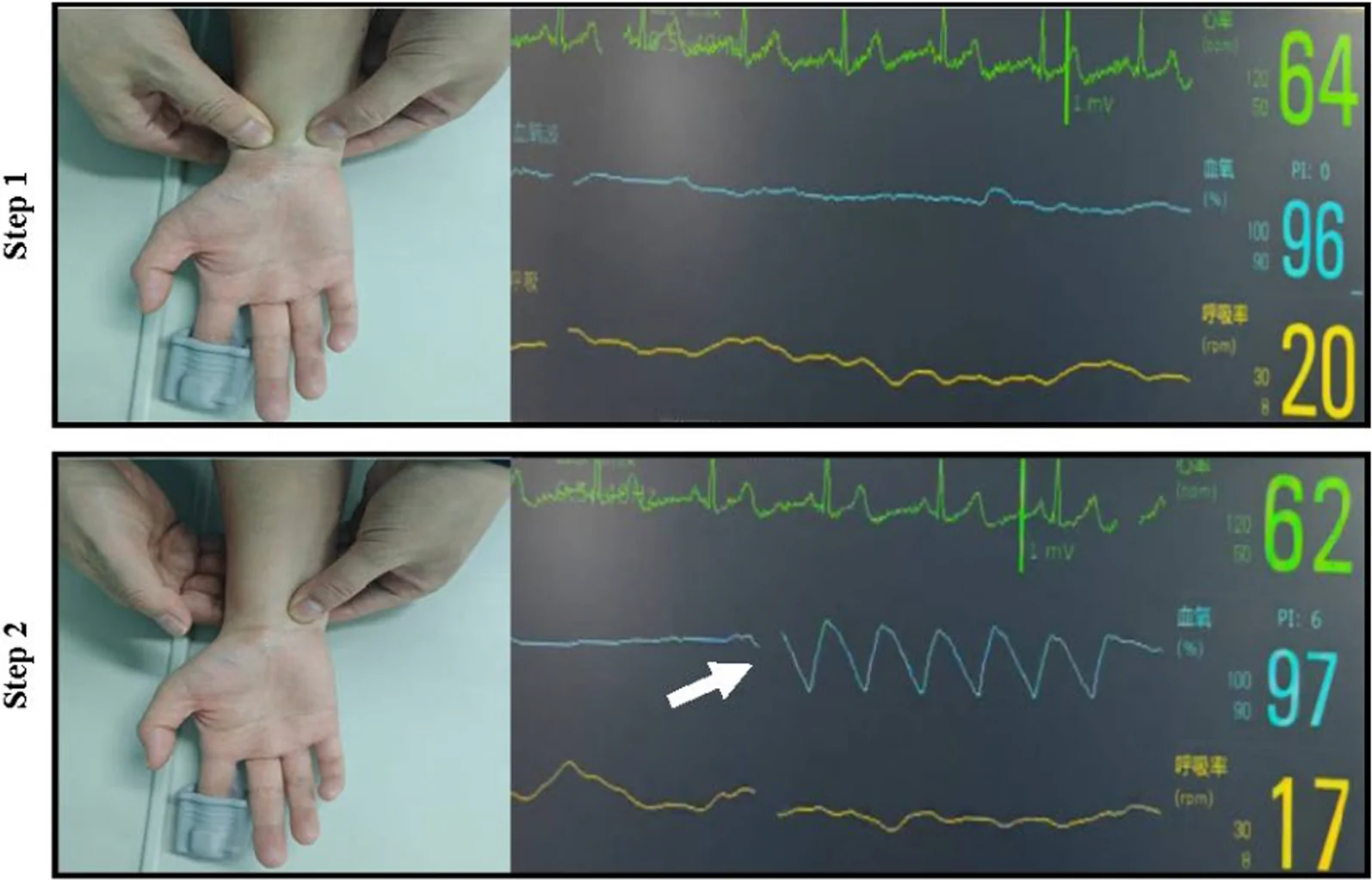
Technique of performance of modified reverse Barbeau’s test (MRBT). The steps of performance of MRBT

While Duplex Doppler ultrasonography (USG) has been extensively considered the gold standard method to evaluate the patency of radial artery after transradial access (TRA), digital plethysmography using MRBT offers distinct advantages. Given considerations of cost, time, and accessibility, MRBT—a more economical and time-efficient approach—has demonstrated diagnostic equivalence to USG in detecting RAO following coronary diagnostic or interventional procedures via TRA [4, 8]. For MRBT test, following sensor placement on the patient’s index finger, continuous plethysmographic waveform monitoring was initiated. Simultaneous compression of both radial and ulnar arteries was maintained until complete waveform obliteration occurred. Subsequent release of radial artery compression prompted waveform evaluation: reappearance within 5 seconds confirmed radial artery patency. Figure 2 illustrates the MRBT procedure and presents a comparative analysis of plethysmographic waveform characteristics between occluded and patent radial arteries.

### Puncture procedure and hemostasis

The procedure was performed as follows: patients were placed supine with arms abducted. Under local anesthesia, radial artery puncture was performed using the Seldinger technique at the site of maximal pulsation. A 0.035-inch non-hydrophilic guidewire was introduced, followed by insertion of a 6F radial sheath. Nitroglycerin (200 μg) and heparin were administered through the sheath.All patients received 5,000 IU heparin for CAG; those undergoing PCI received an additional weight-adjusted dose to a total of 100 U/kg, with activated clotting time (ACT) maintained at 250–300s.Hemostasis was achieved using a TR Band™ device (Terumo Corp., Tokyo, Japan) with a stepwise decompression protocol. Compression was maintained for 2 hours post-CAG and 4 hours post-PCI. Approximately 3-5 mL of air was withdrawn every 60 minutes in the absence of active bleeding. If bleeding occurred, compression was reapplied at 10-minute intervals until hemostasis was confirmed before resuming gradual decompression [9, 10].

### Definitions

RAO was defined as a persistent Type D plethysmographic pattern (no waveform recovery after radial artery release) on the reverse Barbeau test performed 24 hours post-procedure.

Radial artery spasm (RAS) is characterized by increased resistance to catheter movement, patient-reported forearm pain, and occasionally, difficulty in catheter or sheath withdrawal.

Multiple puncture attempts were defined as three or more separate needle insertion attempts at the same or adjacent sites required to achieve successful radial artery cannulation.

### Statistical methods

Statistical analyses were conducted utilizing SPSS version 26 statistical software. Continuous variables were presented as median (IQR), while categorical data were expressed as percentages. Comparisons between groups for continuous variables were performed using the Mann–Whitney U test, whereas chi-square tests were applied for categorical variable analysis. Univariate logistic regression screened variables associated with RAO (*p* < 0.1 for multivariate entry); multivariate logistic regression (backward stepwise) identified independent predictors. The odds ratio (OR) point estimate was accompanied by a 95% two-sided confidence interval (CI), and the model incorporated all potential confounders in variable selection. Additionally, two-tailed hypothesis tests were applied, with statistical significance defined as p<0.05. A nomogram was built based on independent predictors, with weighted scores assigned per regression coefficients to quantify individual RAO risk.R software (v4.2.1, packages: rms, rmda, pROC) was used to construct the nomogram and complete model performance validation: ROC curve (AUC) for discrimination, calibration curve combined with Hosmer-Lemeshow test for calibration assessment, and decision curve analysis (DCA) for evaluating clinical net benefit.

## Results

### Baseline patient characteristics between the two groups

Of the 561 patients with suspected angina pectoris who underwent right transradial CAG or PCI, 49 (8.7%) developed RAO. Table 1 demonstrates distinct clinical profiles in RAO patients compared to non-RAO group, including higher female predominance (66.7% vs. 37.2%), lower median body weight (65.0 vs. 70.0kg). The RAO group also showed significantly higher rates of prior coronary artery disease (66.7% vs. 40.2%), along with elevated CHA2DS2-VASc scores (median 4.0 vs. 3.0). Moreover, the RAO group had higher proportions of prior PCI (43.8% vs. 27.5%, p = 0.027) and prior transradial angiography (50% vs. 27.1%, p = 0.001). In terms of medication use, the RAO group had significantly higher rates of P2Y12-receptor inhibitor (29.2% vs. 16.2%, p = 0.038), β-Blockers (37.5% vs. 21.8%, p = 0.023), and nitrates use (22.9% vs. 11.3%, p = 0.035).

**Table 1:** Comparision of baseline characteristics

**Variables**	**nonRAO (n=513)**	**RAO (n=48)**	***p*-value**
Age (year)	65 [57-72]	67 [60-74]	0.19
Female sex [n (%)]	191 (37.2%)	32 (66.7%)	**<0.001**
Body weight (kg)	70 [62-78]	65 [59-70]	**0.013**
Body mass index (kg/m2)	25.51 [23.44-27.68]	25.37 [23.41-27.73]	0.804
Active smoking [n (%)]	157 (30.6%)	10 (20.8%)	0.211
Diabetes mellitus [n (%)]	133 (25.9%)	18 (37.5%)	0.119
Arterial hypertension [n (%)]	273 (53.2%)	28 (58.3%)	0.597
Hypercholesterolemia [n (%)]	81 (15.8%)	8 (16.7%)	1.000
Prior stroke or TIA [n (%)]	112 (21.8%)	12 (25%)	0.746
Coronary artery disease [n (%)]	206 (40.2%)	32 (66.7%)	**<0.001**
Impaired LV function (EF<55%) [n (%)]	25 (4.9%)	4 (8.3%)	0.299
Prior myocardial infarction [n (%)]	34 (6.6%)	4 (8.3%)	0.555
Prior PCI [n (%)]	141 (27.5%)	21 (43.8%)	**0.027**
Prior transradial angiography [n (%)]	139 (27.1%)	24 (50%)	**0.001**
Atrial fibrillation [n (%)]	27 (5.3%)	2 (4.2%)	1.000
Mean CHA2DS2-VASc score	3 [2–4]	4 [3–5]	**0.019**
Aspirin [n (%)]	243 (47.4%)	27 (56.2%)	0.305
P2Y12-receptor inhibitor [n (%)]	83 (16.2%)	14 (29.2%)	**0.038**
β-Blockers [n (%)]	112 (21.8%)	18 (37.5%)	**0.023**
ACEI/ARB [n (%)]	170 (33.1%)	17 (35.4%)	0.873
Calcium channel blockers [n (%)]	114 (22.2%)	9 (18.8%)	0.709
Nitrates [n (%)]	58 (11.3%)	11 (22.9%)	**0.035**
Statins [n (%)]	260 (50.7%)	29 (60.4%)	0.255
Oral anticoagulant [n (%)]	20 (3.9%)	0 (0%)	0.402

Data are presented as median [interquartile range] or number (percent). P-values by either Mann-Whitney U or Chi-square test

TIA, transient ischemic attack; LV, left ventricle; CAG, coronary angiography; PCI, percutaneous coronary intervention; ACEI, angiotensin converting enzyme inhibitor; ARB, angiotensin receptor inhibitor.

No significant differences were noted in age, BMI, smoking, diabetes, hypertension, or use of aspirin/ACEI/ARB/statin/oral anticoagulants between groups.

### Laboratory and periprocedural characteristics

Table 2summarizes laboratory and perioperative parameters. Among laboratory parameters, the RAO group had significantly higher creatine kinase (98 U/L vs. 83 U/L, *p* = 0.009), platelet count (251 ×10⁹/L vs. 220 ×10⁹/L, *p* = 0.005), and lower hemoglobin (134 g/L vs. 140 g/L, *p* = 0.014) compared with the non-RAO group. No significant differences were found in lipid profiles, glucose, renal function, inflammatory markers (e.g., C-reactive protein), or coagulation parameters (e.g., fibrinogen) between groups.

**Table 2:** Comparison of laboratory testresults and periprocedural characteristics

**Variables**	**nonRAO(n = 513)**	**RAO(n=48)**	***p*-value**
Triglycerides (mmol/L)	1.38 [1.01-1.97]	1.48 [0.98-1.72]	0.642
Fasting ghucose (mmol/L)	5.27 [4.8-6.42]	5.48 [4.7-7.12]	0.749
Total cholesterol (mmol/L)	4.09 [3.37-5.09]	3.8 [3.19-4.34]	0.153
LDL-C (mmol/L)	2.07 [1.55-2.87]	1.94 [1.4-2.45]	0.315
HDL-C(mmol/L)	1 [0.87-1.17]	1.1 [0.92-1.31]	0.083
Lipoprotein(a)(mg/L)	219 [117-434]	229 [129.75-519.75]	0.651
sdLDL-C(mmol/L)	0.8 [0.56-1.18]	0.7 [0.48-0.94]	0.059
eGFR (mL/min/1.7373m2)	120 [99.52-120]	120 [103.11-120]	0.815
Serum creatinine (μmol/L)	61 [52-74]	58.5 [47-71.25]	0.146
Blood urea (mmol/L)	5.72 [4.77-6.89]	5.43 [4.4-6.93]	0.184
Uric acid(μmol/L)	291 [247-350]	266 [203.25-354.25]	0.102
Creatine kinase(U/L)	83 [56-108]	98 [69-162.5]	**0.009**
Platelet count (10⁹/L)	220 [188-262]	251 [202-282]	**0.005**
Hemoglobin(g/L)	140 [130-152]	134 [122.75-146]	**0.014**
Hematocrit value(%)	42.8 [39.9-45.9]	42 [38.88-45.23]	0.071
Platelet distribution width (%)	12.1 [10.8-14.2]	11.85 [10.57-14.15]	0.653
Red blood cell distribution width (%)	12.7 [12.2-13.2]	12.6 [12.3-12.95]	0.899
Neutrophil count (10⁹/L)	3.87 [3.03-4.89]	3.7 [2.7-4.87]	0.808
Lymphocyte count (10⁹/L )	1.9 [1.5-2.4]	1.9 [1.5-2.4]	0.525
Fibrinogen(g/L)	2.77 [2.32-3.3]	2.83 [2.38-3.46]	0.365
Albumin (g/L)	43.9 [41-46]	44.55 [42.08-46.7]	0.211
FAR	6.29 [5.31-7.69]	6.36 [5.66-8.07]	0.290
C-reactive protein (mg/L)	0.8 [0.5-3]	0.95 [0.5-3.02]	0.819
Multiple puncture attempts[n (%)]	33 (6.4%)	21 (43.8%)	**<0.001**
Catheterization time(>15min)[n (%)]	13 (2.5%)	13 (27.1%)	**<0.001**
Radiation time (sec)	405 [173-718]	479.5 [229.75-732.25]	0.288
Operation time(min)	43 [31-70]	45 [32-70]	0.891
Arterial spasm[n (%)]	17 (3.3%)	16 (33.3%)	**<0.001**

Data are presented as median [interquartile range] or number (percent). p values by either Chi-square test or Mann-Whitney U. Abbreviations: LDL -C, low-density lipoprotein cholesterol; HDL -C, high-density lipoprotein cholesterol; sdLDL-C, smalldense low-density lipoprotein cholesterol; eGFR, estimated glomerular filtration rate; FAR, fibrinogen-to-albumin ratio

For periprocedural characteristics, the RAO group had markedly higher rates of multiple puncture attempts (43.8% vs. 6.4%, p < 0.001), puncture duration >15 minutes (27.1% vs. 2.5%, p < 0.001), and radial arterial spasm (33.3% vs. 3.3%, p < 0.001). Radiation time and total operation time showed no significant between-group differences (p = 0.288 and p = 0.891, respectively).

### Univariable and multivariable predictors of RAO

Univariate and multivariate logistic regression analyses were performed to identify independent predictors of RAO (Table 3). In the univariate analysis, female sex (OR = 3.37, *p* < 0.001), prior coronary artery disease (OR = 2.98, *p* = 0.001), CHA2DS2-VASc score (OR = 1.31, *p* = 0.010), platelet count (OR = 1.01,* p* = 0.005), hemoglobin (OR = 0.98, *p* = 0.017), multiple puncture attempts (OR = 11.31, *p* < 0.001), puncture duration >15 min (OR = 14.29,* p* < 0.001), and radial arterial spasm (OR = 14.59, *p* < 0.001) were significantly associated with an increased risk of RAO.

**Table 3:** Univariate and multivariate analyses for RAO

**Variable**	**Univariate**		**Multivariate**	
	**OR (95%CI)**	***P*-value**	**OR (95%CI)**	***P*-value**
Age	1.01 (0.99–1.04)	0.313		
Female sex	3.37 (1.83–6.45)	0.000	2.18 (0.98–5.05)	0.061
Body mass index	0.99 (0.91–1.08)	0.825		
Active smoking	0.6 (0.28–1.18)	0.161		
Diabetes mellitus	1.71 (0.91–3.15)	0.087	1.33 (0.61–2.78)	0.462
Arterial hypertension	1.23 (0.68–2.27)	0.497		
Hypercholesterolemia	1.07 (0.45–2.25)	0.874		
Prior stroke or TIA	1.19 (0.58–2.31)	0.613		
Coronary artery disease	2.98 (1.62–5.7)	0.001	2.26 (1.12–4.68)	**0.025**
Impaired LV function (EF<55%)	1.77 (0.51–4.83)	0.307		
Prior myocardial infarction	1.28 (0.37–3.4)	0.654		
Atrial fibrillation	0.78 (0.12–2.73)	0.743		
CHA2DS2-VASc score	1.31 (1.07–1.6)	0.010	1.01 (0.78–1.3)	0.951
Aspirin	1.43 (0.79–2.62)	0.241		
Triglycerides	1.09 (0.92–1.27)	0.210		
Total cholesterol	0.88 (0.68–1.11)	0.293		
LDL-C	1.07 (0.8–1.35)	0.624		
Creatine kinase	1 (1–1)	0.299		
Platelet count (×10⁹/L)	1.01 (1–1.01)	0.005	1 (1–1.01)	0.383
Hemoglobin	0.98 (0.96–1)	0.017	1 (0.98–1.02)	0.924
Multiple puncture attempts	11.31 (5.77–22.18)	0.000	3.06 (1.12–7.85)	**0.024**
Catheterization time >15 min	14.29 (6.13–33.49)	0.000	3.48 (1.04–11.4)	**0.040**
Radiation time (sec)	1 (1–1)	0.339		
Operation time (min)	1 (0.99–1.01)	0.825		
Arterial spasm	14.59 (6.73–31.75)	0.000	5.18 (1.93–13.4)	**0.001**

After adjusting for potential confounding factors in the multivariate model, prior coronary artery disease (adjusted OR = 2.26, 95% CI: 1.12–4.68, p = 0.025), multiple puncture attempts (adjusted OR = 3.06, *p* = 0.024), puncture duration >15 min (adjusted OR = 3.48, *p* = 0.040), and radial arterial spasm (adjusted OR = 5.18, *p* = 0.001) remained independent predictors of RAO, whereas female sex, CHA2DS2-VASc score, platelet count, and hemoglobin no longer exhibited statistical significance (all *p* > 0.05).

### Performance of the predictive model for RAO

**Fig. 3:**
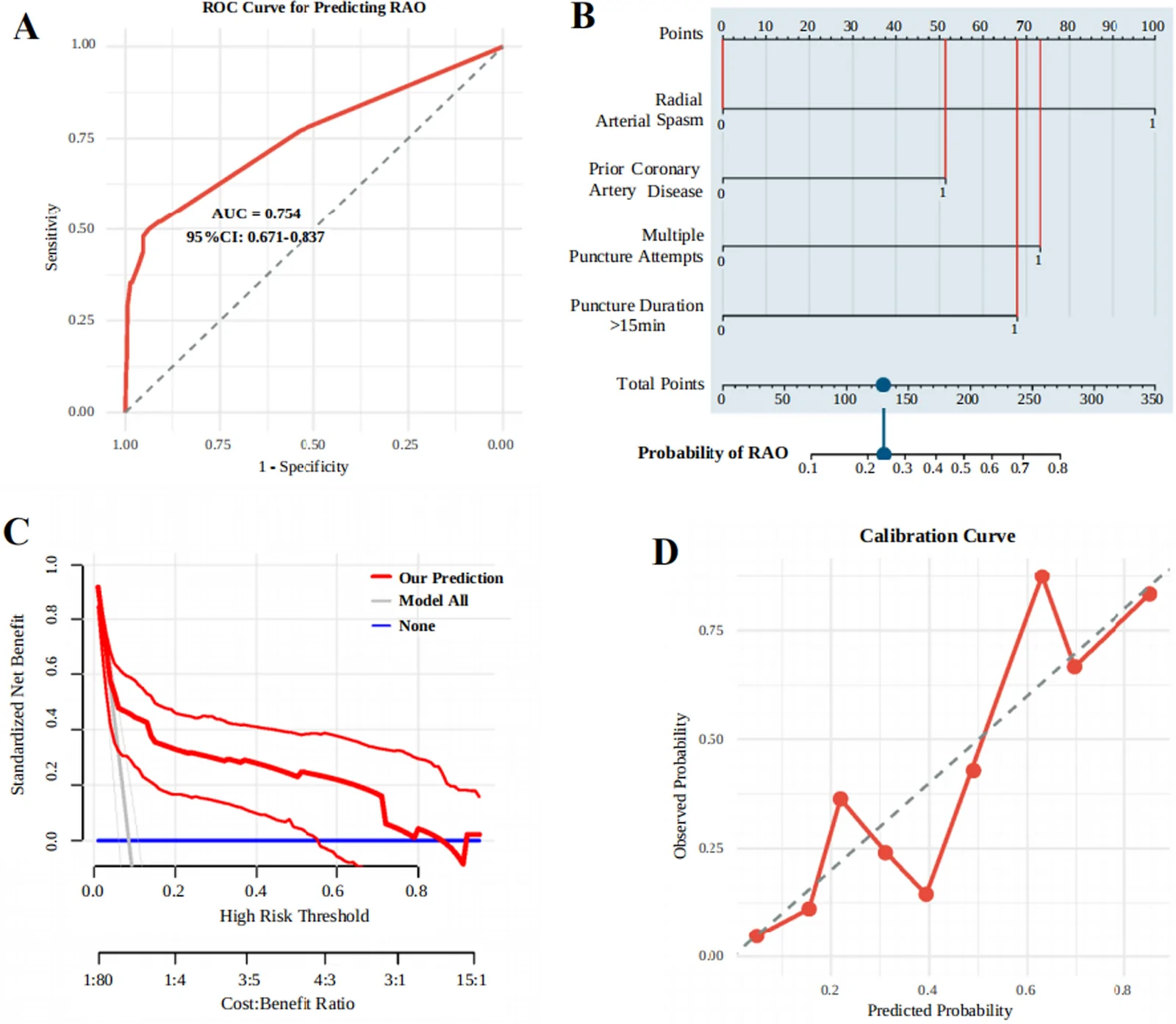
Performance of the Predictive Model for RAO. (**A**) Receiver operating characteristic (ROC) curve ofthe predictive model for RAO. (**B**) Nomogram for predicting the individual risk of RAO. (**C**) Decision curveanalysis (DCA) of the nomogram for predicting. (**D**) Calibration curve of the nomogram for predicting

The predictive model constructed based on the independent predictors (prior coronary artery disease, multiple puncture attempts, puncture duration>15 min and radial arterial spasm) exhibited good discriminative ability for RAO. The area under the ROC curve (AUC) was 0.754 (95% CI: [0.671-0.837], P < 0.001; Fig. 3A).

A nomogram was developed to visually quantify the individual risk of RAO (Fig. 3B). Each independent predictor was assigned a specific score. The total score was calculated by summing the scores of all predictors, and the corresponding probability of RAO could be directly read from the nomogram. For example, a patient with prior coronary artery disease, multiple puncture attempts, puncture duration >15 min and radial arterial spasm would have a total score of 130, corresponding to an approximate 24.5% risk of developing RAO. Decision curve analysis was conducted to evaluate the clinical utility of the nomogram (Fig. 3C). The DCA curve showed that when the threshold probability of RAO was between 0% and 80%, using the nomogram to guide clinical decision-making resulted in a higher net benefit compared with the "treat all" or "treat none" strategies. This indicated that the nomogram could effectively identify patients who would benefit from preventive interventions for RAO, while avoiding unnecessary interventions in low-risk patients, thus demonstrating favorable clinical applicability.

Subsequently, the calibration curve was generated to assess the agreement between the predicted probability of RAO by the nomogram and the actual observed incidence (Fig. 3D). The calibration curve showed that the predicted probabilities were closely aligned with the actual observed outcomes across the entire range of risk levels, with the Hosmer-Lemeshow test yielding a p-value of 0.536 (> 0.05), indicating no significant deviation between predicted and observed risks. This confirmed that the nomogram had good calibration and could accurately predict the absolute risk of RAO in clinical practice.

## Discussion

In the present study, we developed and validated a prediction model for radial artery occlusion (RAO) following transradial coronary procedures, focusing on patient-related and procedural risk factors. The overall incidence of RAO in our cohort was 8.7%, which is consistent with real-world clinical practice and previously published findings [11]. We ultimately identified four independent predictors of RAO: prior coronary artery disease, multiple puncture attempts, puncture duration >15 min, and radial artery spasm, and constructed a prediction nomogram with favorable clinical utility.

Prior coronary artery disease was identified as the strongest patient-related independent predictor of RAO in our model. This finding is biologically plausible: patients with prior coronary artery disease present with a persistent hypercoagulable phenotype mediated by activated inflammatory pathways. Elevated circulating malondialdehyde, RhoA/ROCK signaling activity, and pro-inflammatory cytokines amplify platelet reactivity and coagulation cascade activation under minor vascular stimulation [12, 13]. Compared with patients without atherosclerotic heart disease, these individuals have higher baseline platelet counts and creatine kinase (CK) levels—both laboratory markers linked to increased RAO risk in univariate and multivariate analyses [14, 15]. Even limited radial intimal denudation from routine cannulation is sufficient to generate occlusive thrombus in this pre-activated thrombotic environment, whereas intact vasculature in patients without prior coronary artery disease tolerates equivalent procedural trauma with far lower occlusion risk [1, 16].

Multiple puncture attempts, puncture duration >15 min, and radial artery spasm were confirmed as independent procedural predictors of RAO, consistent with prior research [14, 17, 18]. Multiple punctures disrupt the vascular endothelium, exposing subendothelial matrix to trigger platelet aggregation and coagulation activation, thereby elevating RAO susceptibility [18]. The tunica media of the radial artery is rich in α-adrenergic smooth muscle receptors, which confer marked vulnerability to mechanical trauma from repeated needle puncture and local tissue irritation. Sustained physical stimulation of the vessel wall at the puncture site induces persistent, severe vascular spasm, which is characterized by sudden stenosis of the arterial lumen [18]. This lesion aggravates intimal injury, disrupts local hemodynamics, and jointly promotes thrombosis [19]. Excessively prolonged compression for hemostasis is a well-documented key contributor to RAO, as over-compression facilitates local thrombus formation [20, 21]. To mitigate this risk, a conventional graded balloon deflation protocol was uniformly adopted for all subjects in the present study [22]; however, due to the lack of standardized hemostasis duration data, this factor was not incorporated into our final model.

Several factors showed significant associations with RAO in univariate analysis but lost independent predictive value after multivariate adjustment. Female sex and lower body weight were associated with higher RAO risk in univariate analysis, which aligns with prior evidence: female patients generally have a smaller radial artery diameter [23], which increases the risk of sheath–artery mismatch, vascular endothelial injury, and subsequent thrombotic occlusion. However, after multivariate adjustment, the effect of female vascular vulnerability was offset by coronary artery disease burden and intraoperative trauma, leading to the loss of independent significance. Similarly, CK levels were significantly higher in the RAO group in univariate analysis but were not retained in the multivariate model. CK is a non-specific marker reflecting skeletal muscle damage and perioperative tissue trauma, with no direct pathophysiological linkage to local radial artery thrombosis; thus, preoperative CK levels cannot independently predict RAO and are not recommended as a preoperative risk screening index in clinical practice.

Compared with our original unadjusted analysis, the final modified model integrated both patient-related and procedural variables, focusing on the four independent predictors of RAO. The prediction nomogram showed good discrimination, calibration, and clinical utility, which may facilitate individualized preoperative risk assessment for RAO. This model offers distinct advantages in clinical practice: it is cost-effective, straightforward to interpret, and can be easily integrated into standard preoperative workflows for patients undergoing transradial coronary procedures.

### Limitations

This study has several important limitations that should be considered when interpreting the results. First, this was a single-center retrospective study, which may limit the generalizability of the findings to broader patient populations. Second, several potential RAO-related factors, such as perioperative blood pressure, hemostatic compression duration, and repeated compression strategies, were not incorporated into our multivariate analysis and warrant further exploration in subsequent research. Third, all patients were treated with a uniform 6F sheath, which does not fully reflect contemporary clinical practice with smaller sheath sizes and may have increased the baseline RAO risk in our cohort. Fourth, the prediction model was developed and internally validated in a single cohort without external validation, and its reproducibility and generalizability need to be confirmed in future prospective multicenter studies. Finally, some potential confounders, such as radial artery diameter, were not available in our dataset, which may have a slight impact on the estimated effect sizes. Although the modified reverse Barbeau test (MRBT) has been validated against duplex ultrasonography, ultrasound remains the reference standard for RAO diagnosis and was not routinely performed in this study.

## Conclusions and future directions

In conclusion, our study demonstrates that prior coronary artery disease, multiple puncture attempts, puncture duration >15 min, and radial artery spasm are independent predictors of RAO following transradial coronary procedures, emphasizing the importance of optimized procedural technique to reduce RAO risk. We constructed and internally validated a novel prediction nomogram with favorable discrimination, calibration, and clinical utility for individualized RAO risk assessment. Future studies should incorporate modifiable procedural and hemostasis-related factors, conduct external validation in multicenter prospective cohorts, and explore the clinical value of this model in guiding personalized perioperative management to reduce the incidence of RAO.

## Supplementary Information

Additional file 1.

## Data Availability

The data used to support the findings of this study are available from the corresponding author upon request.

## References

[1] Tsigkas G, Papanikolaou A, Apostolos A, et al. Preventing and managing radial artery occlusion following transradial procedures: strategies and considerations. J Cardiovasc Dev Dis. 2023;10:283. 10.3390/jcdd10070283 PMC10380353 37504539

[2] Hahalis GN, Leopoulou M, Tsigkas G, et al. Multicenter randomized evaluation of high versus standard heparin dose on incident radial arterial occlusion after transradial coronary angiography: the SPIRIT OF ARTEMIS study. JACC Cardiovasc Interv. 2018;11:2241–50. 10.1016/j.jcin.2018.08.009 30391389

[3] Lavi S, Mehta SR, Bajwa R, et al. Short durations of radial hemostatic device after diagnostic transradial cardiac catheterization: the PRACTICAL-2 randomized trial. Canadian J Cardiol. 2021;37(2):276–83. 10.1016/j.cjca.2020.04.017 32335132

[4] Pancholy SB, Patel V, Pancholy SA, et al. Comparison of diagnostic accuracy of digital plethysmography versus duplex ultrasound in detecting radial artery occlusion after transradial access. Cardiovasc Revasc Med. 2021;27:52–6. 10.1016/j.carrev.2020.07.025 32855082

[5] Petroglou D, Didagelos M, Chalikias G, et al. Manual versus mechanical compression of the radial artery after transradial coronary angiography: the MEMORY multicenter randomized trial. JACC Cardiovasc Interv. 2018;11:1050–8. 10.1016/j.jcin.2018.03.042 29880098

[6] Wagener JF, Rao SV. Radial artery occlusion after transradial approach to cardiac catheterization. Curr Atheroscler Rep. 2015;17:489. 10.1007/s11883-015-0489-6 25651786

[7] Avdikos G, Karatasakis A, Tsoumeleas A, et al. Radial artery occlusion after transradial coronary catheterization. Cardiovasc Diagn Ther. 2017;7:305–16. 10.21037/cdt.2017.03.14 PMC5440258 28567356

[8] Alberti M, Bucca G, Somenzi A, et al. Radial artery occlusion after a radial access procedure: pilot study comparing eco doppler and inverse barbeau test assessments. Assist Inferm Ric. 2021;40:213–20. 10.1702/3743.37260 35138303

[9] Xu D, Liu Y, Xu C, Liu X, Chen Y, Feng C, et al. Factors affecting radial artery occlusion after right transradial artery catheterization for coronary intervention and procedures. Ther Clin Risk Manag. 2023;19:525–33. 10.2147/TCRM.S403410 PMC10305768 37388675

[10] Ma K, Li J, Shen G, Zheng D, Xuan Y, Lu Y, et al. Development and validation of a risk nomogram model for predicting contrast-induced acute kidney injury in patients with non-ST-elevation acute coronary syndrome undergoing primary percutaneous coronary intervention. Clin Interv Aging. 2022;17:65–77. 10.2147/CIA.S349159 PMC8801515 35115770

[11] Rashid M, Kwok CS, Pancholy S, et al. Radial artery occlusion after transradial interventions: a systematic review and meta-analysis. J Am Heart Assoc. 2016;5:e002686. 10.1161/JAHA.115.002686 PMC4859386 26811162

[12] Dokumacioglu E, Duzcan I, Iskender H, et al. RhoA/ROCK-1 signaling pathway and oxidative stress in coronary artery disease. Braz J Cardiovasc Surg. 2022;37(2):212–8. 10.21470/1678-9741-2020-0525 PMC9054146 34236817

[13] Vilahur G, Fuster V. Interplay between platelets and coagulation: from protective haemostasis to pathological arterial thrombosis. Eur Heart J. 2025;46(5):413–23. 10.1093/eurheartj/ehae776 39673717

[14] Didagelos M, Pagiantza A, Papazoglou AS, Moysidis DV, Petroglou D, Daios S, et al. Incidence and prognostic factors of radial artery occlusion in transradial coronary catheterization. J Clin Med. 2024;13:3276. 10.3390/jcm13113276 PMC11173088 38892987

[15] Xie B, Chen N, Teng Y, Feng H. Multivariate analysis and individualized nomogram construction for predicting radial artery occlusion risk after transradial intervention. BMC Surg. 2026;26:171. 10.1186/s12893-026-03536-5 PMC12955333 41622164

[16] Khalid A, Mautong H, Ahmed K, et al. Incidence and predictors of early and late radial artery occlusion after percutaneous coronary intervention and coronary angiography: a systematic review and meta-analysis. J Clin Med. 2024;13(19):5882. 10.3390/jcm13195882 PMC11477189 39407942

[17] Jian W, Cai R, Qi B, Li Q. Reduced radial artery occlusion in transradial cerebral angiography: key predictive factors and preventive measures from a single-center study of 543 patients. Med Sci Monit. 2024;30:e944297. 10.12659/MSM.944297 PMC11302386 39037961

[18] Buturak A, Gorgulu S, Norgaz T, Voyvoda N, Sahingoz Y, Degirmencioglu A, et al. The long-term incidence and predictors of radial artery occlusion following a transradial coronary procedure. Cardiol J. 2014;21:350–6. 10.5603/CJ.a2013.0128 24142678

[19] Coghill EM, Johnson T, Morris RE, Megson IL, Leslie SJ. Radial artery access site complications during cardiac procedures, clinical implications and potential solutions: the role of nitric oxide. World J Cardiol. 2020;12:26–34. 10.4330/wjc.v12.i1.26 PMC6952722 31984125

[20] Takamatsu S, Kagiyama N, Sone N, Tougi K, Yamauchi S, Yuri T, et al. Impact of radial compression protocols on radial artery occlusion and hemostasis time in coronary angiography. Cardiovasc Interv Ther. 2023;38:202–9. 10.1007/s12928-022-00896-6 PMC10020254 36478329

[21] Karadayi A, Unal NA, Mutlu EG, Engiz BK, Akkoca A, Varol S. Effects of exposure to radiofrequency at 2.45 GHZ on structural changes associated with lipid peroxidation in prepubertal rat testicular tissue. Eur J Ther. 2023;29:846–55.

[22] Pancholy SB, Patel TM. Effect of duration of hemostatic compression on radial artery occlusion after transradial access. Catheter Cardiovasc Interv. 2012;79:78–81. 10.1002/ccd.22963 21584923

[23] JSSVCN, Sameer K. Relationship between radial artery diameters with incidence of radial artery occlusion after transradial coronary catheterization: an observational cohort study. Scholars J Appl Med Sci. 2024;12:1652–6.

