# Supplementary material for: Risk factors of radial artery occlusion following via transradial coronary catheterization
Source: J Cardiothorac Surg. 2026 Jul 29;21:844. doi: 10.1186/s13019-026-04608-6 (PMC13700124; doi:10.1186/s13019-026-04608-6)
Supplement: Supplementary file 1 [file 13019_2026_4608_MOESM1_ESM.pdf]

Document No.: AF-35/06.2
Version: 2023-10-01

Medical Ethics Committee of the Affiliated Hospital of Xuzhou Medical University

Ethic Review Form

Approved No.	XYFY2025-KL408-01				
Project Name	Establishment and verification of nomogram model for predicting the risk of radial artery occlusion in patients after percutaneous coronary intervention via radial artery				
Research Institute	The Affiliated Hospital of Xuzhou Medical University				
Department	Department of Cardiovascular Medicine	PI	Hui feng	Title	associate chief physician
Review Category	scientific research project		Review Mode	Summary procedure review	
Date	2025-8-15		Location	commissioner's office	
Applicant Name	The Affiliated Hospital of Xuzhou Medical University				
Document	1、research approach 2、application for informed consent-free 3、resume of the main researcher, members of the research team and responsibilities 4、statement of economic interests of researcher 5、commitment letter of scientific research integrity				

Review Comments

Comply with the Ministry of Health "Protocols for the Ethical Review of Biomedical Research Involving Human) (2016), National Health Commission, Ministry of Education, Ministry of Science and Technology, State Administration of traditional Chinese Medicine "Ethical Review measures for Human Life Sciences and Medical Research" , NMPA "Drug Clinical Trial Quality Management Standards" (2020), "Medical Device Clinical Trial Quality Management Standards" (2022), "Clinical Application of Medical Technology" Management Protocols" (2018), WMA "Declaration of Helsinki" and CIOMS "International Ethical Guidelines for Human Biomedical Research" and the Ethical Principles of Clinical Trial Management Guidelines (ICH-GCP). The Ethics Committee have reviewed and approved this research to performed based on the clinical research protocol, informed consent form, and materials for recruiting subjects.

Voting: 1 votes agree; 1 votes agreed after making necessary amendments (☐ quick review, ☐ regular review); 1 votes disagree, 1 people withdrew due to conflicts of interest.

Please follow the GCP principles, the ICH-GCP guidelines, and the protocol approved by the Ethics Committee to conduct clinical research to protect the health and rights of the subjects.

The Ethics Committee will follow up and review the approved project until the end of the study. The specific requirements are：<br>① If the main investigator is changed during the research process, the applicant is required to submit an amendment review application for any changes to the clinical research protocol, informed consent form, recruitment materials, etc.<br>②Please follow the annual/periodical follow-up review frequency set by the Ethics Committee. Applicants should submit a research progress report one month before the deadline; the sponsor should submit a summary report of the research progress of each center to the group leader's Ethics Committee; in case of any possibilities that significantly affect the progress of the experiment or increase the risk of the subjects, Applicants are requested to submit a written report to the Ethics Committee in a timely manner.<br>③In the event of serious adverse events and unanticipated adverse drug reactions, applicants are requested to submit a written report to the Ethics Committee in a timely manner.<br>④The study included subjects who did not meet the inclusion criteria or met the exclusion criteria, met the requirements for suspension of the trial but did not allow the subjects to withdraw from the study, given the wrong treatment or dose, given the combined drugs prohibited by the protocol, etc., did not follow the protocol to conduct the research; In case of violation of GCP principles, which may cause adverse effects on the rights and interests of subjects/health and the scientific nature of the research, the sponsor/inspector/investigator is requested to submit a report on the violation of the protocol.<br>⑤ Applicants who suspend or terminate clinical research early, please submit the suspension/termination report in time.<br>⑥ After completion of the clinical research, applicants are requested to submit a research completion report.

Annual/regular follow-up review frequency	12 months
Validated period	2 years
Signature	Guojun Cui
Issue date	2025-8-19
Ethics Committee	Medical Ethics Committee of the Affiliated Hospital of Xuzhou Medical University（Stamp）

Address： 99 West Huai hai Road，221000， Tel：0516-85802291
